# Overexpression of ZEB2‐AS1 promotes epithelial‐to‐mesenchymal transition and metastasis by stabilizing ZEB2 mRNA in head neck squamous cell carcinoma

**DOI:** 10.1111/jcmm.14318

**Published:** 2019-04-04

**Authors:** Pengfei Diao, Han Ge, Yue Song, Yaping Wu, Jin Li, Zhongwu Li, Jianrong Yang, Yanling Wang, Jie Cheng

**Affiliations:** ^1^ Jiangsu Key Laboratory of Oral Disease Nanjing Medical University Nanjing China; ^2^ Department of Oral and Maxillofacial Surgery Affiliated Stomatological Hospital, Nanjing Medical University Nanjing China

**Keywords:** EMT, head neck squamous cell carcinoma, long noncoding RNA, ZEB2, ZEB2‐AS1

## Abstract

The long noncoding RNAs (lncRNAs) have been increasingly appreciated as key players underlying tumourigenesis and hold great potentials as prognostic biomarkers and therapeutic targets. However, their roles in head neck squamous cell carcinoma (HNSCC) have remained incompletely known. Here, we sought to reveal the oncogenic roles and clinical significance of a tumour‐associated lncRNA, zinc finger E‐box binding homeobox 2 antisense RNA 1 (ZEB2‐AS1), in HNSCC. ZEB2‐AS1 was aberrantly overexpressed in a fraction of HNSCC samples. Its overexpression significantly associated with large tumour size, cervical node metastasis and reduced overall and disease‐free survival. Antisense oligonucleotides (ASO)‐mediated ZEB2‐AS1 depletion markedly inhibited cell proliferation, migration and invasion while triggered apoptosis in HNSCC cells in part via modulating ZEB2 mRNA stability. Enforced overexpression of ZEB2 largely attenuated the phenotypic changes resulted from ZEB2‐AS1 inhibition except the impaired cell proliferation. In addition, ZEB2‐AS1 was required for TGF‐β1‐induced epithelial‐mesenchymal transition (EMT) in vitro. Significantly reduced tumour growth and lung metastasis were observed in ZEB2‐AS1‐depleted cells in HNSCC xenograft animal models. Taken together, our findings reveal that overexpression of ZEB2‐AS1 associates with tumour aggressiveness and unfavourable prognosis by serving as a putative oncogenic lncRNA and a novel prognostic biomarker in HNSCC.

## 1 INTRODUCTION

Head neck squamous cell carcinoma (HNSCC) is the sixth most common malignancy worldwide with about 500 000 new cases diagnosed each year and also remains as one leading cause of cancer‐related death.^1^ Although comprehensive treatment regime including surgical resection, radiotherapy and chemotherapy has been established for years, the overall 5‐year survival rate of HNSCC patients has not been significantly improved over the past decades.^2^ A large fraction of patients has been definitively diagnosed with diseases in advanced stages, thus precluding early and timely clinical management of these malignancies. Several prognostic factors like clinical stage, invasive depth, status of surgical margins as well as cervical node involvement have been identified to be significantly associated with local recurrence, distant metastasis and patient survival.^3^ However, these prognostic biomarkers for HNSCC are still far from optimal. These unmet challenges in HNSCC diagnosis, treatment and prognostic prediction highlight the urgent need to identify optimal biomarkers and druggable targets.

The past decades have witnessed tremendous progress in genomic profiling and functional interrogation of noncoding RNAs in human cancer. Among these cancer‐related noncoding RNA species, long noncoding RNAs (lncRNAs) are a class of transcripts longer than 200 nucleotides which have been increasingly appreciated as key players underlying tumourigenesis.^4^ Accumulating evidence has suggested that aberrantly deregulated lncRNAs have linked to cancer initiation and overgrowth, metastatic spreading and therapeutic resistance.^4^, ^5^ These lncRNAs usually modulate gene expression at the chromatin organization, transcriptional or posttranscriptional levels as decoys, guides or scaffolds.^6^ Noticeably, natural antisense transcript (NAT), a specific class of lncRNA, is transcribed from the opposite DNA strand to other transcripts and overlaps in part with sense RNA, which has lineage‐specific or cancer‐specific expression manner.^7^, ^8^ A number of molecular mechanisms including transcription‐related modulation, RNA‐DNA interaction and RNA‐RNA interaction have been proposed for antisense‐mediated regulation of cognate sense RNA.^9^, ^10^ To date, genomic transcriptional profiling and function dissection of lncRNA in HNSCC remains far from complete. Several lncRNA such as HOTAIR, H19 and DLEU1 have been revealed to facilitate initiation and progression of HNSCC and their overexpression serve as diagnostic and prognostic biomarkers.^11^, ^12^, ^13^ However, quite few NATs have been identified as key oncogene or tumour suppressor in HNSCC thus far. The lncRNA zinc finger E‐box binding homeobox 2 antisense RNA1 (ZEB2‐AS1) has attracted our attentions as it was highly expressed in several solid cancers like hepatocellular carcinoma, lung cancer and pancreatic cancer. Moreover, it usually promoted cell proliferation, invasion and EMT while inhibited apoptosis in diverse cancer contexts.^14^, ^15^, ^16^, ^17^ These findings suggested that ZEB2‐AS1 was an oncogenic NAT with incompletely delineated roles in human cancer. However, the expression and biological roles of ZEB2‐AS1 underlying HNSCC remain largely unexplored yet.

In the present study, we sought to determine the expression pattern of ZEB2‐AS1 in primary HNSCC and uncover its roles during HNSCC tumourigenesis by loss‐of‐function assay and xenograft animal model.

## 2 MATERIALS AND METHODS

### 2.1 Patients and tissue specimens

A total of 71 pairs of fresh HNSCC and adjacent non‐tumour mucosa were included from patients treated in Department of Oral and Maxillofacial Surgery, Affiliated Stomatological Hospital, Nanjing Medical University from January 2011 to December 2017. All patients were diagnosed as primary HNSCC without any prior treatments and underwent radical resection at our institution. All tissue samples were snap‐frozen in liquid nitrogen and stored in at −80°C until RNA extraction. Written informed consent was obtained from these patients. Detailed information including epidemiologic, clinical, pathological and follow‐up data for each patient was available. This study protocol was reviewed and approved by the Research Ethics Committee of Nanjing Medical University.

### 2.2 HNSCC cell lines and cell culture

A panel of cell lines including normal human oral keratinocytes (HOK) and HNSCC cell lines FaDu, Cal27, HN4, HN6, SCC4 and SCC25 were used. HOK, FaDu, Cal27, SCC4 and SCC25 cells were purchased from American Type Culture Collection (ATCC) and authenticated by short tandem repeat (STR) profiling. HN4 and HN6 cells were kindly gifted from Dr Wantao Chen (Shanghai JiaoTong University). All cells were routinely tested for mycoplasma at regular intervals. Cancerous cells were grown in DMEM/F12 (Invitrogen) supplemented with 10% FBS (Gibco) and penicillin‐streptomycin (1%), and maintained at 37°C in a 5% CO_2_‐humidified incubator. For TGF‐β1‐induced EMT cellular model in vitro, cells were treated with recombinant human TGF‐β1 (rhTGF‐β1, 10 ng/mL, R&D Systems) for indicated time‐points. Subsequently, changes of cell morphology and EMT‐relevant markers were monitored accordingly.

### 2.3 Antisense oligonucleotides (ASO) biosynthesis, small interfere RNAs, lentiviral construct and transfection

The antisense oligonucleotides specifically targeting human ZEB2‐AS1 (Gene ID: 100303491) including ASO‐ZEB2‐AS1‐92 (5'‐A*C*A*C*T*T*C*G*C*G*G*C*T*T*C*T*T*C*A*T‐3'), ASO‐ZEB2‐AS1‐265 (5'‐G*C*T*C*A*G*G*G*C*G*C*T*T*C*A*A*T*T*A*T‐3') and ASO‐ZEB2‐AS1‐316 (5'‐A*T*G*C*C*C*T*A*A*G*A*T*G*C*A*G*C*T*C*C‐3') (*indicative of phosphorothioate) were designed and purchased from Sangon Biotech (Shanghai, China). This phosphorothioate modification was aimed to enhance stability and efficiency of ASO both in vitro and in vivo.^18^, ^19^ For ZEB2 knockdown, two independent small interference RNAs (siRNA) were designed and synthesized. The sequence was listed as follows: siZEB2‐1: CTAGACTTCAATGACTATAAA; siZEB2‐2: CTGTACTTTCCTTTCGCTATT, which targeted coding region and 3' UTR of human ZEB2. Transfection of ASOs (final concentration: 50 nmol/L ASOs) or siRNAs (final concentration: 100 nmol/L) was performed with lipofectamine RNAiMAX (Life Technologies) according to the manufacturer's instructions. Cells were harvested for further analyses 48 hours after transfection.

For ZEB2 overexpression and rescue experiments, the human ZEB2 overexpression construct tagged with FLAG was generated by inserting its full‐length cDNA template into lentiviral plasmid pLenti CMV‐GFP‐Puro and verified by direct sequencing. Lentiviral particles were routinely prepared as we previously reported.^20^ The efficiency of ZEB2 overexpression construct was confirmed by western blot following cell infection in vitro. For ZEB2 rescue experiments, cells were initially treated with siRNAs targeting ZEB2‐AS1 alone. Twenty‐four hours later, these cells were infected with ZEB2 overexpressing lentivirus or empty vector for another 24 hours and then harvested for further analyses.

### 2.4 RNA isolation and quantitative reverse transcription‐quantitative PCR (qRT‐PCR)

Total RNA was extracted from cells or tissues with TRIzol reagent (Invitrogen) and then subjected to reverse transcription and PCR reactions using PrimeScript^TM ^RT‐PCR kit (Takara). The primers used were listed as follows: for ZEB2‐AS1, 5'‐ACAAAGATAGGTGGCGCGTG‐3' (forward) and 5'‐GCATGAAGAAGCCGCGAAGTGT‐3' (reverse); for ZEB2, 5'‐TGAGGATGACGGTATTGC‐3' (forward) and 5'‐ATCTCGTTGTTGTGCCAG‐3' (reverse); for 18S rRNA, 5'‐ACACGGACAGGATTGACAGA‐3' (forward) and 5'‐GGACATCTAAGGGCATCACA‐3' (reverse); for U6, 5'‐CTCGCTTCGGCAGCACA‐3' (forward) and 5'‐AACGCTTCACGAATTTGCGT‐3' (reverse).

To characterize the location of ZEB2‐AS1 in HSNCC cells, cytoplasmic and nuclear fractions of FaDu and Cal27 cells were prepared and collected using the PARIS^TM^ Kit (Life Technologies, Carlsbad, CA, USA). 18S rRNA was used as the endogenous cytoplasmic control. U6 small nuclear RNA was used as the endogenous nuclear control. The nuclear and cytoplasmic RNA was further analysed by qRT‐PCR. Relative mRNA or lncRNA expression was quantified as compared to internal control GAPDH or U6 using comparative CT method.

### 2.5 CCK‐8, colony formation and cell apoptosis assay

Cell proliferation and viability were assessed by absorbance using CCK‐8 cell viability assay (Cell Counting Kit‐8, Dojindo, Japan). Control and ASO‐ZEB2‐AS1‐treated HNSCC cells were seeded into the 96‐well plates at an initial density of 1 × 10^3^ cells per well. CCK‐8 solution (10 μL/well) was added to the cells at specified time‐points. For colony formation assay, 1000 single cells pretreated with ASO‐ZEB2‐AS1 were seeded into 6‐well plates and allowed to grow for 12 days. After stained with crystal violet, the colonies were visualized under microscope, photographed and counted. For cell apoptosis assay, **c**ells were treated with trypsin (Gbico), resuspended as single‐cell suspension and then stained with Annexin V: PE Apoptosis Detection Kit (BD Bioscience).

### 2.6 Cell migration and invasion assay

Cell migration and invasion assays in vitro were performed with wound healing and transwell chambers (8‐μm pore size, Corning) with Matrigel (BD Pharmingen) pre‐coating as we previously reported.^20^, ^21^

### 2.7 Western blot analysis

Cells in culture flasks or plates were lysed in ice‐cold buffer containing protease inhibitor cocktail (Roche). Equal amounts of protein samples were loaded and separated by SDS‐PAGE and transferred to PVDF membranes (Millipore) followed by non‐fat milk or BSA blocking. These blots were incubated at 4°C overnight with primary antibodies against ZEB2 (1:1000 dilution; Proteintech, 14026‐1‐AP), Cleaved‐PARP (1:1000 dilution; Cell Signaling, #9541), N‐cadherin (1:1000 dilution; Cell Signaling, #13116), E‐cadherin (1:1000 dilution; Cell Signaling, #14472), Vimentin (1:1000 dilution; GeneTex, GTX100619), Snail (1:1000 dilution; Cell Signaling, #3879) and GAPDH (1:5000, MB001, Bioworld) followed by incubation with horseradish peroxidase (HRP)‐conjugated secondary antibodies at room temperature. Immunoreactive bands on the blots were detected by ECL chemiluminescence kit (Millipore).

### 2.8 Cellular immunofluorescence assay

For immunofluorescence assays, cells were seeded on glass coverslips 24 hours prior to experiment and then fixed with 4% paraformaldehyde, washed thoroughly with PBS and permeabilized in Triton X‐100 (0.1% in PBS) for 1 hour. Then cells were blocked with 3% BSA for 30 minutes followed by incubation with primary antibodies against E‐cadherin (1:200 dilution) and Vimentin (1:150 dilution) overnight respectively. Immunofluorescence was visualized under a Zeiss fluorescence microscope or confocal microscope.

### 2.9 HNSCC xenograft model and tail vein injection lung metastasis mouse model

All experiments involving animal subjects were in accordance with the institutional animal welfare guidelines and approved by Institutional Animal Care and Use Committee of Nanjing Medical University.

Six‐week‐old female nu/nu mice were obtained and maintained in a specific pathologic‐free environment. Stable ZEB2‐AS1 knockdown cancer cells (2 × 10^6^) suspended in total 100 μL PBS and diluted Matrigel (1:1) were inoculated subcutaneously on the right flanks (six animals per experimental group). Tumour incidence and growth were monitored after injection. Tumour diameters were measured by calipers every 3 days when tumour masses were identified. Tumour volume is calculated as follows: volume = a × b^2^/2, where a and b are defined as the longest diameter and shortest diameter respectively. Final tumour weight was measured upon animals were killed. Tumour samples were further processed for routine H&E staining, immunohistochemical staining, etc. For the lung metastasis model, 1 × 10^6^ FaDu cells were injected into nu/nu mice via the tail vein. After 10 weeks, the whole lungs were resected and photographed. Lung tissues were sectioned and stained with H&E and CK5/6 (Kit‐0018, Maxim, China) antibody. Lung metastatic lesions were determined and quantified under a microscope (double blinded).

### 2.10 Statistical analysis

All quantitative data in the present study were shown as mean ± SD from two or three independent experiments and compared with Student's *t* test or ANOVA with Bonferroni post hoc test unless otherwise specified. Paired *t* test was utilized to measure the abundance of ZEB2‐AS1 or ZEB2 in paired HNSCC and adjacent non‐tumour samples. The potential associations between ZEB2‐AS1 expression and various clinicopathological parameters were evaluated by chi‐squared test. The survival rates of patients were estimated using Kaplan‐Meier method and compared with Log‐rank test. *P *< 0.05 (two‐sided) were considered statistically significant. All statistical analyses were performed with GraphPad Prism 7 or SPSS 22.0 software.

## 3 RESULTS

### 3.1 Overexpression of ZEB2‐AS1 correlated with aggressive clinicopathological parameters in HNSCC

Previous studies have revealed up‐regulated lncRNA ZEB2‐AS1 in bladder, lung and pancreatic cancers and its overexpression associated with malignant clinicopathological features.^14^, ^16^, ^17^ To investigate the biological functions of ZEB2‐AS1 and its significance in HNSCC, we initially measured its expression via qRT‐PCR in 71 pairs of freshly collected HNSCC samples and adjacent non‐tumour mucosa. In brief, these patients (42 males and 29 females) were 30‐82 (median 63) years old and with median follow‐up duration 55 months (range 6‐84 months). Until last follow‐up, 39 (54.9%) patients remained alive and also disease‐free, seven (9.9%) still alive but with local recurrences and/or cervical nodal metastases, whereas 25 patients (35.2%) died with disease recurrence, metastasis or unknown reasons.

**Figure 1:**
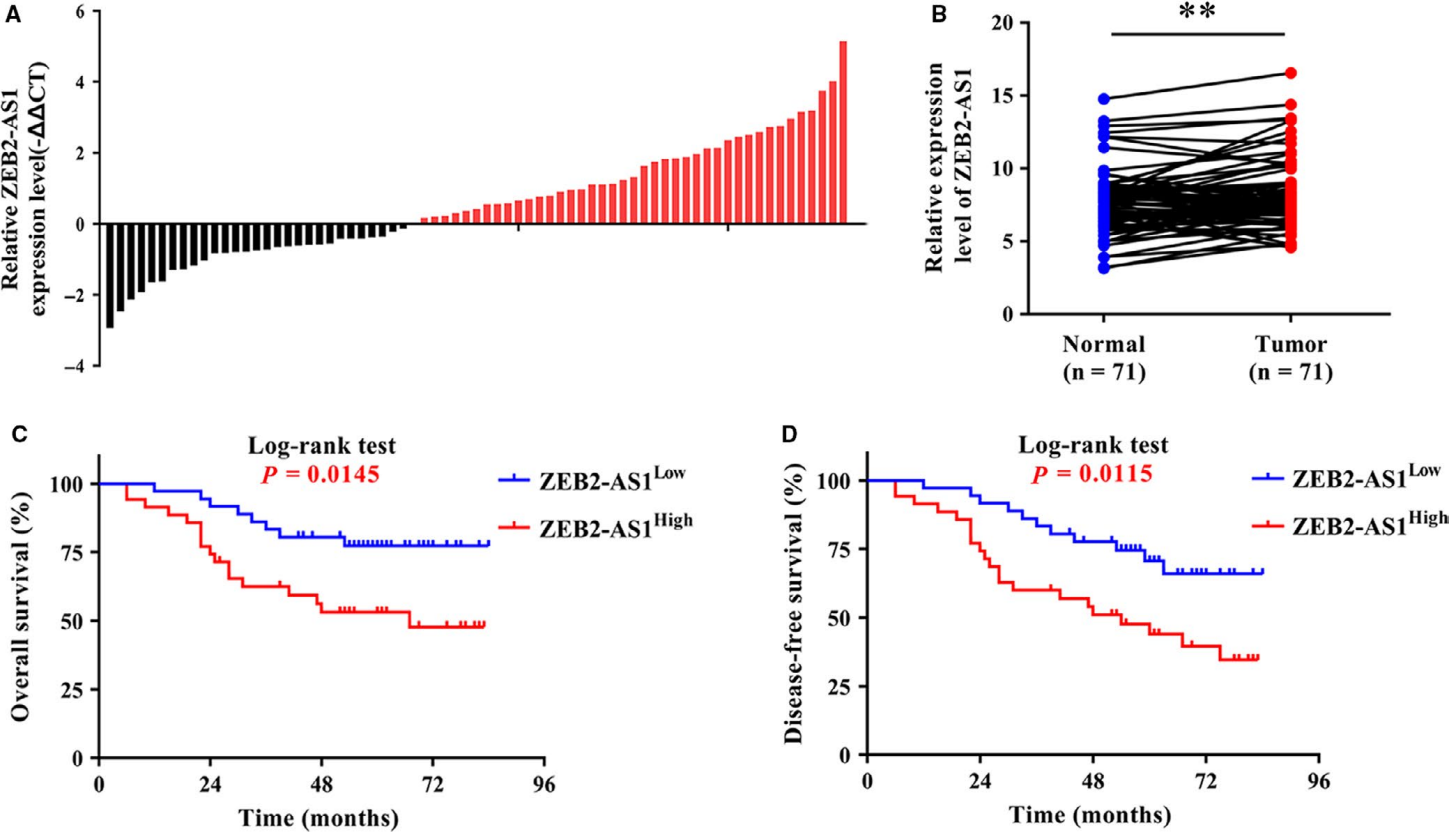
Overexpression of ZEB2‐AS1 in HNSCC tissues associates with cancer aggressiveness and reduced survival in HNSCC. A,B, The relative expression of ZEB2‐AS1 (log2‐transformed) was measured by qRT‐PCR and compared between 71 fresh HNSCC samples and paired normal counterparts. C,D, Overall survival (C) and disease‐free survival (D) analyses of patients with high or low expression of ZEB2‐AS1 were estimated by Kaplan‐Meier method and compared with Log‐rank test. **P < 0.01, Paired *t* test or Log‐rank test

The fold changes of ZEB2‐AS1 (HNSCC versus paired adjacent normal mucosa) in samples derived from each patient were shown in Figure 1A. Quantification data revealed that ZEB2‐AS1 was significantly up‐regulated in a large fraction of samples (Figure 1B). To identify the possible relationship between ZEB2‐AS1 expression and clinicopathological parameters in HNSCC, these patients were stratified into subgroups with high (n = 35) or low (n = 36) ZEB2‐AS1 expression when the median of ZEB2‐AS1 expression was utilized as cut‐off. As detailed in Table S1, overexpression of ZEB2‐AS1 was significantly associated with larger tumour size (*P* = 0.0287) and cervical node metastasis (*P* = 0.0321). We failed to identify the associations between ZEB2‐AS1 and other demographic or clinicopathological parameters. Moreover, as shown in Figure 1C,D, results from Kaplan‐Meier analyses indicated that patients with higher ZEB2‐AS1 expression had inferior overall survival (OS) and disease‐free survival (DFS) than those with lower ZEB2‐AS1 expression (*P* = 0.0145, 0.0115, Log‐rank test). More importantly, our univariate and multivariate Cox regression assays both revealed that status of ZEB2‐AS1 expression was an independent prognostic factor for HNSCC survival (Table S2), strongly suggesting its potential as a novel prognostic biomarker.

### 3.2 ZEB2‐AS1 knockdown inhibited cell proliferation and induced apoptosis in HNSCC cells

**Figure 2:**
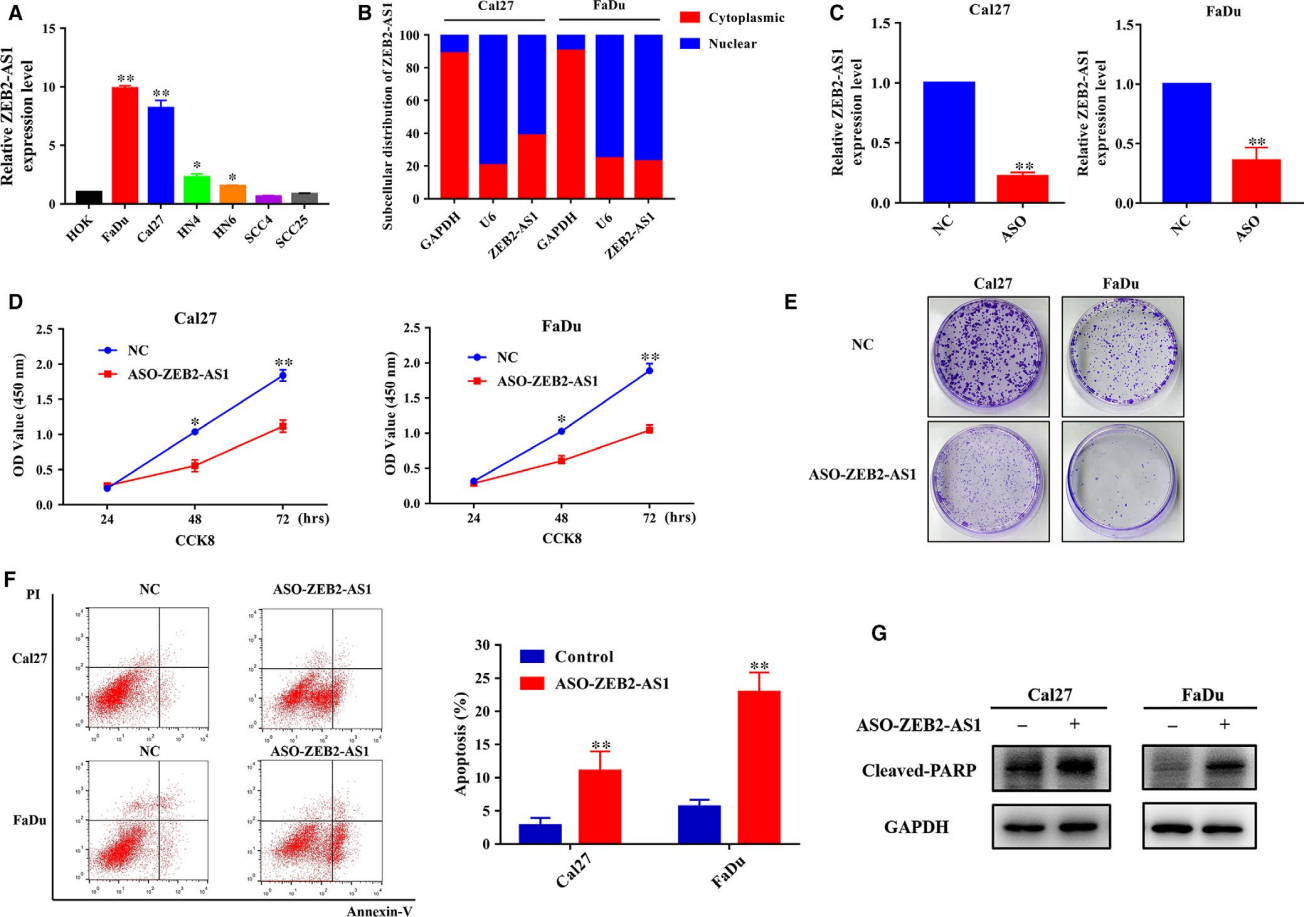
ZEB2‐AS1 knockdown inhibits cell proliferation and triggers apoptosis in HNSCC cells. A, The expression levels of ZEB2‐AS1 in normal human oral keratinocytes (HOK) cell and HNSCC cell lines were measured by qRT‐PCR. B, The cytoplasmic and nuclear distribution of ZEB2‐AS1 RNA in Cal27 and FaDu cells were determined by subcellular fractions and qRT‐PCR (cytoplasmic, red; nuclear, blue). GAPDH served as the cytoplasmic internal control. U6 served as the nuclear internal control. C, The expression change of ZEB2‐AS1 was measured by qRT‐PCR in Cal27 and FaDu cells transfected ASO‐ZEB2‐AS1. D, Cell proliferation was remarkably suppressed as measured by CCK‐8 viability assay upon ZEB2‐AS1 knockdown. E, The ability of colony formation was significantly inhibited in ZEB2‐AS1‐depleted cells (transfected with ASO‐ZEB2‐AS1) as compared to control. F, Increased percentages of apoptotic cell were evident following ZEB2‐AS1 knockdown as assayed by Annexin V‐PI staining. G, Increased expression of the apoptotic marker Cleaved‐PARP was found in ASO‐ZEB2‐AS1‐treated Cal27 and FaDu cells. Data shown here are mean ± SD from three independent experiments, *P < 0.05, **P < 0.01, Student's *t* test or ANOVA analyses

Next, we set out to determine the biological roles of ZEB2‐AS1 in HNSCC by loss‐of‐function assay. We firstly measured the ZEB2‐AS1 and ZEB2 expression in HNSCC cells including FaDu, Cal27, HN4, HN6, SCC4 and SCC25 cells by qRT‐PCR. As displayed in Figure 2A, Cal27 and FaDu cells had relatively higher endogenous expression of ZEB2‐AS1, which was selected for further experiments. RNA fractionation of nuclear and cytoplasmic fractions of Cal27 and FaDu followed by qRT‐PCR revealed that ZEB2‐AS1 expression was enriched in the nucleus and much less in the cytoplasm (Figure 2B). To delineate the roles of ZEB2‐AS1 in HNSCC, we reduced ZEB2‐AS1 expression by transfecting three independent ZEB2‐AS1 antisense oligonucleotides 92, 265 and 316 (ASO‐ZEB2‐AS1) into Cal27 and FaDu respectively. As shown in Figure S1, although three ASO significantly reduced the ZEB‐AS1 expression in both cells, the ASO‐316 showed the highest potency and then was selected for following experiments. This ASO‐316 (hereafter named as ASO‐ZEB2‐AS1 for simplicity) robustly reduced endogenous ZEB2‐AS1 in both Cal27 and FaDu cells (Figure 2C). Subsequently, we monitored the phenotypic changes upon ZEB2‐AS1 depletion in vitro. Cell proliferation was significantly inhibited in both cells upon ZEB2‐AS1 knockdown as demonstrated by the data from CCK‐8 assay (Figure 2D) and colony formation assay (Figure 2E) as compared to the control. In addition, results derived from flow cytometric assay revealed that the proportions of apoptotic cells in ASO‐ZEB2‐AS1‐treated cells were significantly increased from 2.8% to 11.0% in Cal27, from 5.6% to 22.9% in FaDu respectively (Figure 2F). In line with this, the expression of apoptosis marker Cleaved‐PARP was markedly elevated in ASO‐ZEB2‐AS1‐treated cells (Figure 2G).

### 3.3 ZEB2‐AS1 knockdown inhibited cell proliferation, migration and invasion in HNSCC cells by stabilizing ZEB2 mRNA

**Figure 3:**
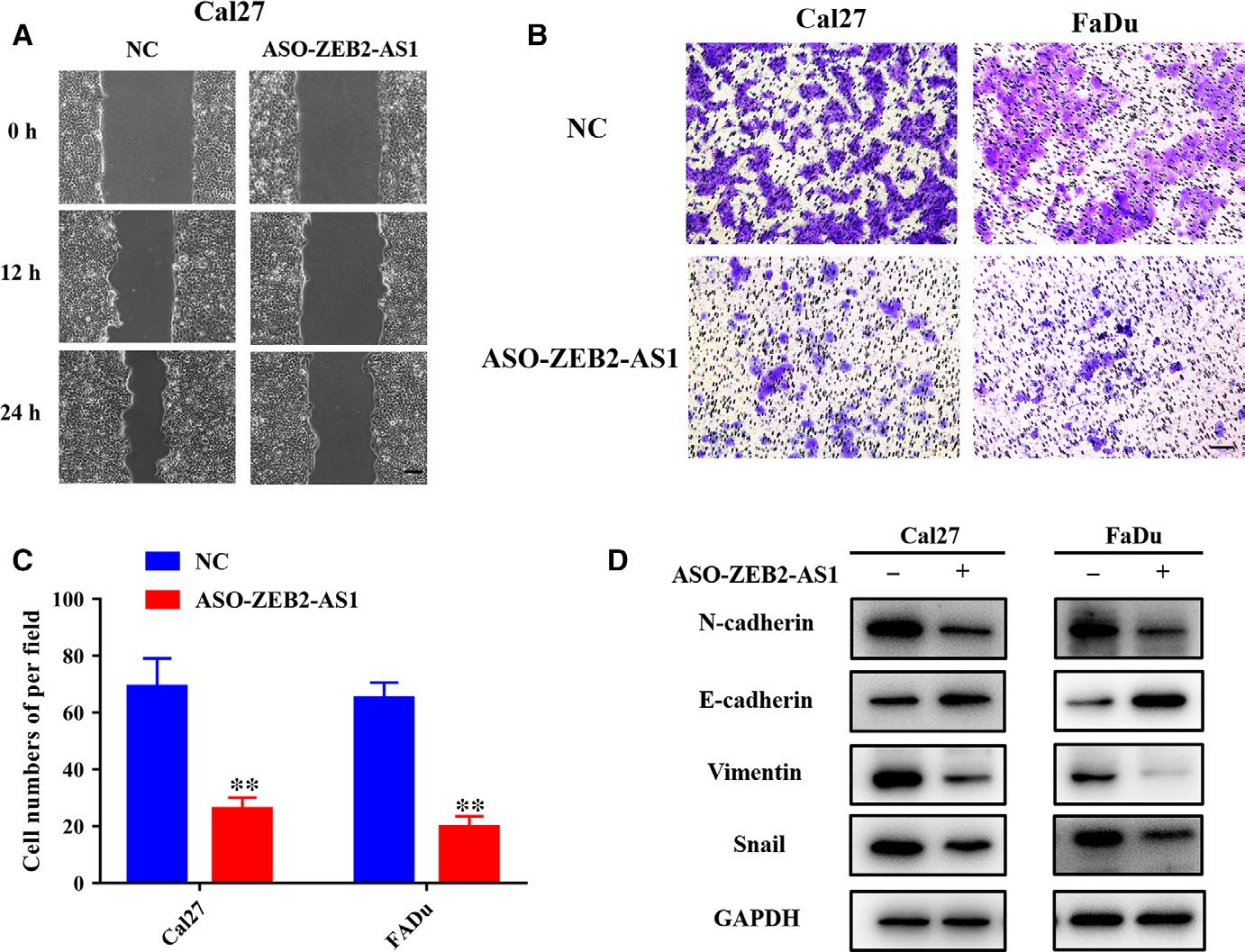
ZEB2‐AS1 knockdown inhibits migration, invasion and EMT in HNSCC cells. A, The migratory ability was significantly impaired in ASO‐ZEB2‐AS1‐transfected Cal27 cells via wound healing assay. Scale bar: 100 μm. B,C, The invasive abilities were significantly reduced in ASO‐ZEB2‐AS1‐transfected Cal27 and FaDu cells in transwell assays respectively. Scale bar: 100 μm. D, The protein abundance of EMT markers E‐cadherin, N‐cadherin, Vimentin and Snail following ZEB2‐AS1 knockdown were assayed by western blot. Data showed here are mean ± SD from three independent experiments. **P < 0.01, Student's *t* test

Next, we performed wound healing and transwell invasion assays to measure cell migratory and invasive potentials of Cal27 and FaDu following ZEB2‐AS1 depletion. As shown in Figure 3A‐C, both migratory and invasive properties of cells were significantly impaired after ZEB2‐AS1 knockdown in HNSCC cells. The levels of EMT‐related markers were further measured by western blot. As shown in Figure 3D, up‐regulation of E‐cadherin and down‐regulation of N‐cadherin, Vimentin and Snail were detected upon ZEB2‐AS1 knockdown, which suggested inhibition of EMT following ASO‐ZEB2‐AS1 introduction in HNSCC cells.

**Figure 4:**
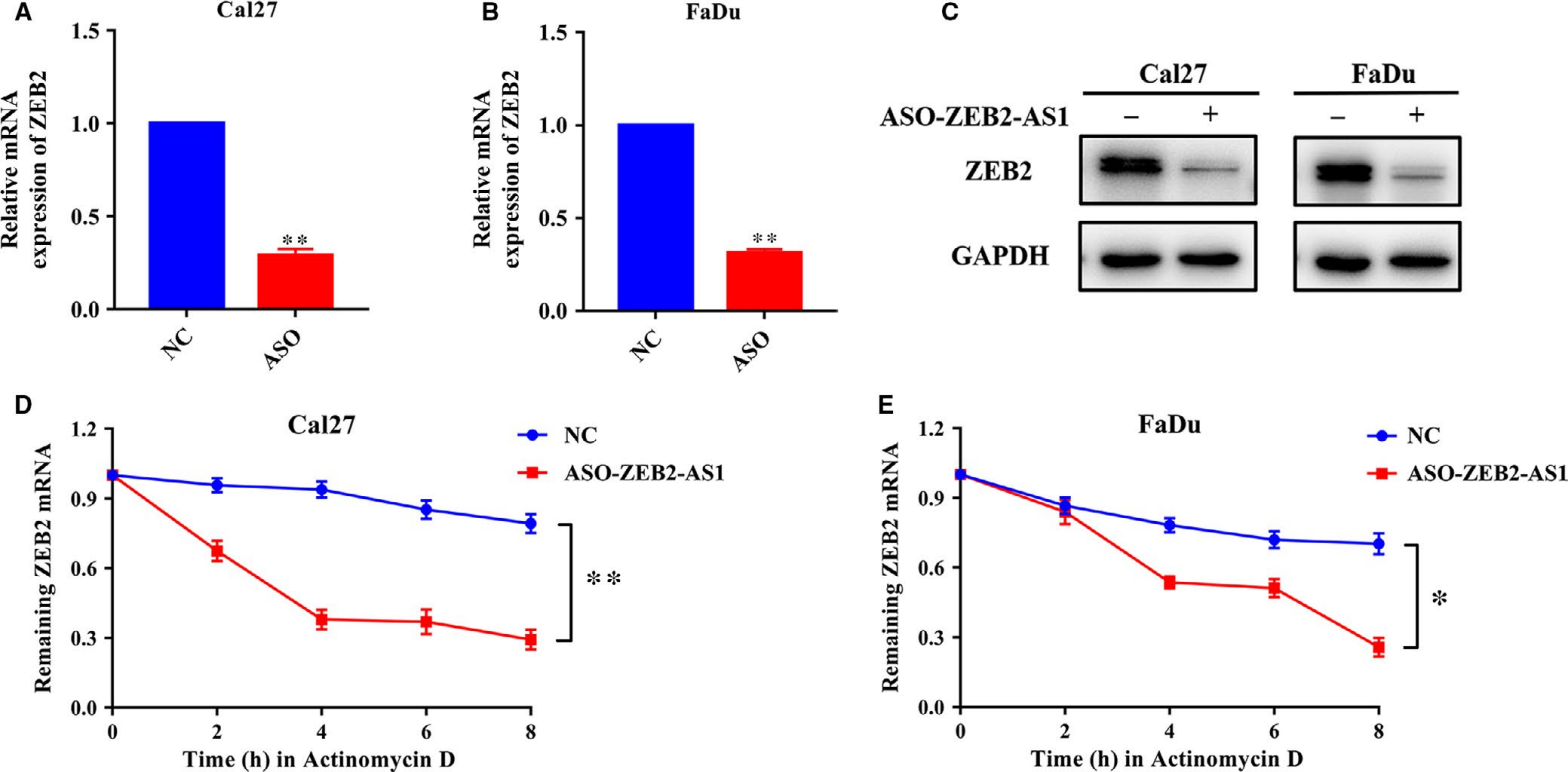
Knockdown ZEB2‐AS1 inhibits ZEB2 mRNA expression and stability in HNSCC cells. A,B, Reduced expression of ZEB2 mRNA was measured via qRT‐PCR in ASO‐ZEB2‐AS1‐treated Cal27 (A) and FaDu (B) cells respectively. C, Down‐regulation of ZEB2 protein was determined by western blot analysis in Cal27 and FaDu cells treated with ASO‐ZEB2‐AS1. D, E: ZEB2‐AS1 knockdown reduced the half‐time of ZEB2 mRNA in cells treated with RNA synthesis inhibitor Actinomycin D (10 µg/mL) in Cal27 (D) and FaDu cells (E). Data shown here are mean ± SD from three independent experiments, *P < 0.05, **P < 0.01, Student's *t* test and ANOVA analyses

**Figure 5:**
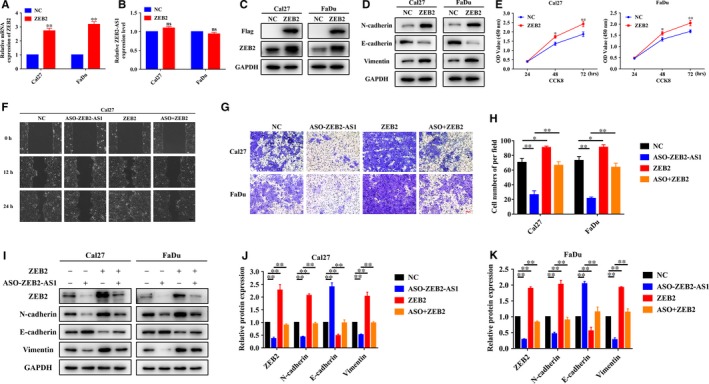
Reintroduction of exogenous ZEB2 largely attenuated the phenotypical changes induced by ZEB2‐AS1 inhibition. A,B, The expression changes of ZEB2 (A) and ZEB2‐AS1 (B) were measured by qRT‐PCR in Cal27 and FaDu cells infected with ZEB2 overexpressing lentivirus. C, Increased ZEB2 protein was confirmed by western blot in Cal27 and FaDu cells infected with ZEB2 overexpressing lentivirus. D, The abundance of EMT markers N‐cadherin, E‐cadherin and Vimentin following ZEB2 overexpression was determined by western blot. E, Cell proliferation was remarkably enhanced following enforced ZEB2 overexpression. F, The migratory abilities were measured via wound healing assay when Cal27 cells were treated with ASO‐ZEB2‐AS1 or in combination with ZEB2 overexpressing lentivirus. Scale bar: 100 μm. G,H, Cell invasiveness was measured via transwell chamber assays when cells were treated with ASO‐ZEB2‐AS1 or in combination with ZEB2 overexpressing lentivirus. Scale bar: 100 μm. I‐K, The protein abundance of EMT‐related markers N‐cadherin, E‐cadherin and Vimentin was measured by when cells were treated with ASO‐ZEB2‐AS1 or in combination with ZEB2 overexpressing lentivirus. Data shown here are mean ± SD from three independent experiments, *P < 0.05, **P < 0.01, Student's *t* test or ANOVA analyses

Considering the pro‐invasive roles of ZEB2‐AS1 in HNSCC cells and the well‐defined roles of ZEB2 in EMT and cancer invasion,^22^, ^23^ we speculated that ZEB2‐AS1 might exert its function by regulating ZEB2. To address this, we firstly performed ZEB2 knockdown assay and monitored the resulting phenotypic changes in vitro. As displayed in Figure S2, siRNA‐mediated ZEB2 knockdown significantly inhibited cell proliferation, migration and invasion in vitro, which largely phenocopied ZEB2‐AS1 inhibition induced by ASOs in HNSCC cells. Thus, these findings suggested that ZEB2‐AS1 might promote HNSCC progression by regulating ZEB2 expression. Indeed, some previous reports have offered vital clues regarding ZEB2‐AS1 in modulating ZEB2 expression in diverse contexts.^24^, ^25^ As shown in Figure 4A‐C, ASO‐ZEB2‐AS1 treatment resulted in significant down‐regulation of ZEB2 mRNA and protein in both HNSCC cells. However, we failed to detect any significant expression changes of ZEB2‐AS1 upon ZEB2 knockdown in vitro (Figure S2B). Next, the chemical actinomycin D which potently inhibits the de novo synthesis of RNA was used to explore whether RNA stability of ZEB2 mRNA was affected by ZEB2‐AS1. Noticeably, depletion of ZEB2‐AS1 resulted in a remarkably decreased half‐life of ZEB2 mRNA in Cal27 and FaDu cells **(**Figure 4D,E). Moreover, we performed the rescue experiments in which ectopic ZEB2 overexpressing lentivirus was introduced into cells with ZEB2‐AS1 inhibition. As shown in Figure 5A‐D, the abundance of ZEB2 mRNA and protein was markedly increased upon ectopic ZEB2 introduction accompanied by expected expression changes of EMT markers, whereas the expression level of ZEB2‐AS1 was largely unaffected. Cells with ZEB2 overexpression had higher proliferation rates than those control cells as assayed by CCK‐8 assay (Figure 5E). Moreover, results from wound healing and transwell invasion assays indicated that reintroduction of ZEB2 attenuated, at least in part, the phenotypic changes induced by ASO‐mediated ZEB2‐AS1 inhibition (Figure 5F‐H). Consistently, the expression changes of EMT‐relevant markers also collaborated changes of these tumourigenic characteristics in vitro (Figure 5I‐K). However, we failed to find that exogenous ZEB2 can reverse the impaired cell proliferation induced by ZEB2‐AS1 depletion in both Cal27 and Fadu cells (Figure S3).

Additionally, our findings from primary HNSCC samples and cell lines revealed higher expression of ZEB2 in HNSCC relative to their non‐tumour counterparts and the positive correlation between ZEB2‐AS1 and ZEB2 in HNSCC primary samples (Pearson correlation analysis, R = 0.9059; *P* < 0.0001; Figure S4A‐C) and cell lines (R = 0.8536, *P* = 0.0029; Figure S4D,E). In addition, data mining and interrogation of TCGA‐HNSCC data set had revealed significant correlation between ZEB2‐AS1 and ZEB2 mRNA in HNSCC (Figure S4F). Collectively, these data support that ZEB2‐AS1 functions by promoting ZEB2 abundance via stabilizing its mRNA in HNSCC cells.

### 3.4 ZEB2‐AS1 is involved in TGF‐β1‐induced EMT in HNSCC

**Figure 6:**
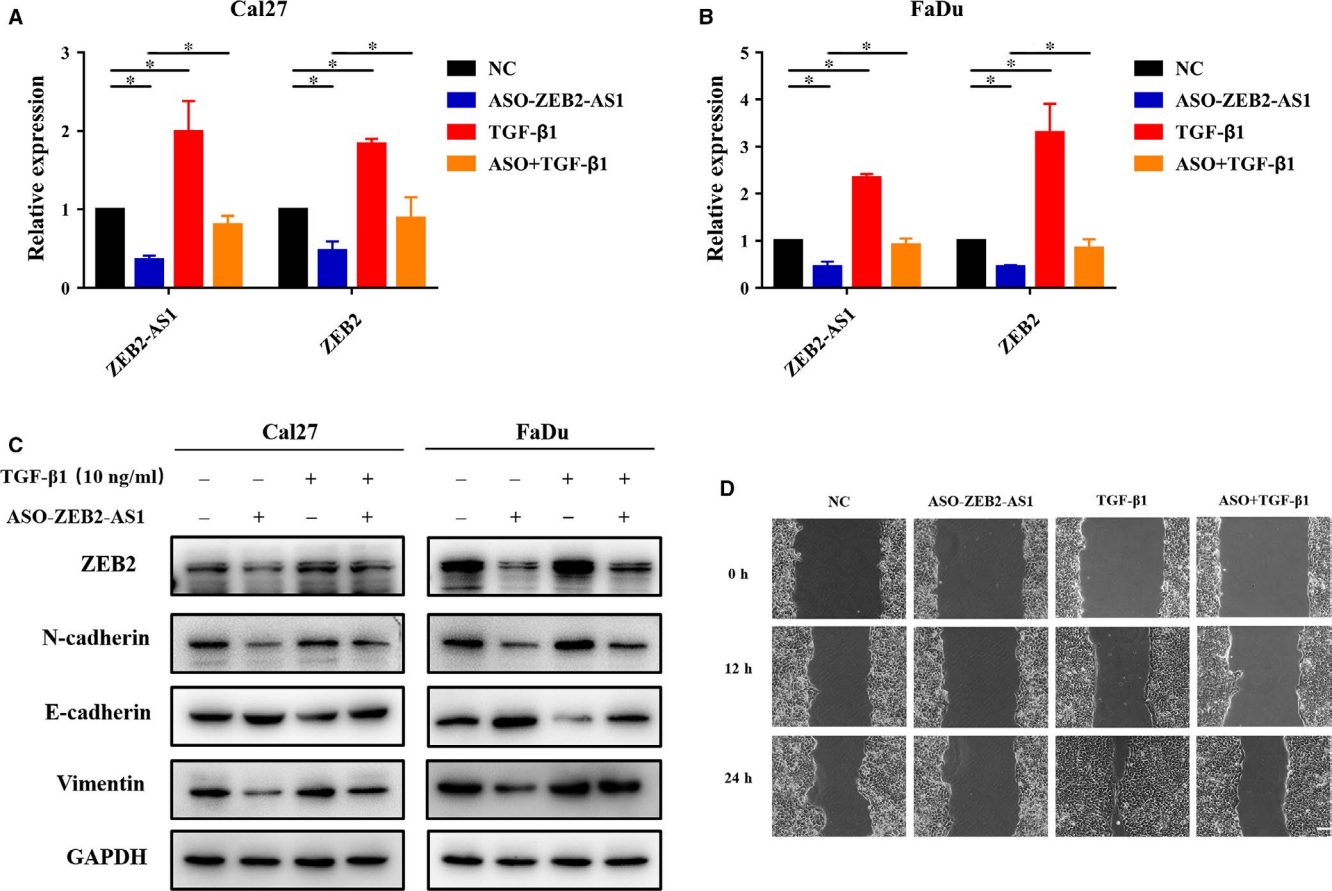
ZEB2‐AS1 is involved for TGF‐β1‐induced EMT in HNSCC cells. A,B, The expression changes of ZEB2‐AS1 and ZEB2 mRNA were measured by qRT‐PCR when Cal27 (A) and FaDu (B) cells were treated with ASO‐ZEB2‐AS1 or in combination with rhTGF‐β1 (10 ng/mL) for 48 h. C, The protein abundance of EMT‐related markers E‐cadherin, N‐cadherin and Vimentin was measured by western blot assays when Cal27 and FaDu cells were treated with ASO‐ZEB2‐AS1 or in combination with rhTGF‐β1 (10 ng/mL) for 48 h. D, The ability of cell migration was measured via wound healing assay when Cal27 cells were treated with ASO‐ZEB2‐AS1 or in combination with rhTGF‐β1 (10 ng/mL) for 48 h. Scale bar: 100 μm. Data showed here are mean ± SD from three independent experiments. *P < 0.05, Student's *t* test analyses

Mounting evidence indicates that EMT‐mediated metastatic spread dictates patients survival in various solid cancers including HNSCC.^23^ Our in vitro loss‐of‐function assays suggested the potential roles of ZEB2‐AS1 involving EMT and invasion of HNSCC. Thus, we next aimed to determine whether ZEB2‐AS1 has the EMT‐inducing role in HNSCC via the conical TGF‐β1‐inducing EMT model.^20^, ^26^ Both ZEB2‐AS1 and ZEB2 were significantly up‐regulated when cells were incubated with rhTGF‐β1 for 48 hours. However, when cells were treated with ASO‐ZEB2‐AS1, rhTGF‐β1 alone or in combination, up‐regulation of ZEB2‐AS1 and ZEB2 mRNA after rhTGF‐β1 exposure was largely abrogated when ASO‐ZEB2‐AS1 was added simultaneously (Figure 6A,B). Consistently, as expected, our western blot and immunofluorescence assays indicated that rhTGF‐β1 treatment resulted in ZEB2, N‐cadherin and Vimentin up‐regulation and E‐cadherin down‐regulation, whereas ZEB2‐AS1 knockdown largely abolished these effects of rhTGF‐β1 (Figure 6C; Figure S5). Moreover, in line with these marker changes, TGF‐β1‐induced enhancement of cell migration was significantly impaired following ZEB2‐AS1 knockdown (Figure 6D).

### 3.5 ZEB2‐AS1 knockdown inhibits tumour growth and metastatic in vivo

**Figure 7:**
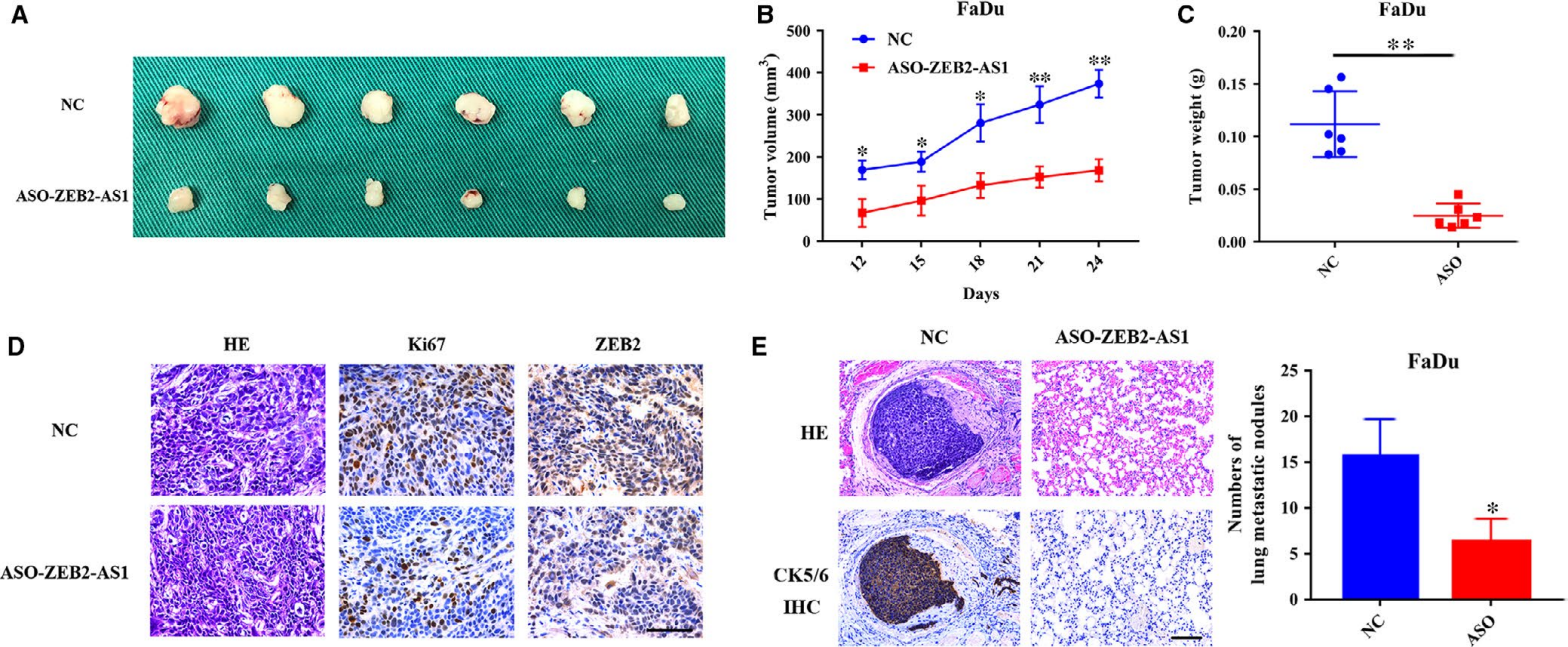
ZEB2‐AS1 depletion impairs tumour growth and metastasis in HNSCC xenograft models. A, Samples formed from FaDu cells treated with ASO‐ZEB2‐AS1 or negative control were shown in a subcutaneous xenograft model. B, Tumour volumes were monitored in xenograft samples derived from FaDu cells with stable ZEB2‐AS1 knockdown or control. C, Final weight of tumour masses harvested from samples derived from FaDu cells with stable ZEB2‐AS1 knockdown or control was compared. D, The expression of ZEB2 and the proliferative marker Ki67 was determined by immunohistochemical staining in xenograft samples derived from FaDu cells with stable ZEB2‐AS1 knockdown or controls. Scale bar: 100 μm. E, H&E staining and CK5/6 immunohistochemical staining of lung metastatic nodules from nude mice injected with FaDu cells with stable ZEB2‐AS1 knockdown or control. Scale bar: 100 μm. *P < 0.05, **P < 0.01, Student's *t* test and ANOVA analyses

To further verify the pro‐tumourigenic role of ZEB2‐AS1 and to explore the therapeutic potential of ZEB2‐AS1 targeting in HNSCC, we established HNSCC xenograft tumour models using FaDu cell inoculated into left flanks of nude mice. When the same amount of cells treated with control ASO (negative control, NC) and ZEB2‐AS1‐ASO was inoculated, the tumours generated from ZEB2‐AS1‐ASO‐treated cells were much smaller with much less volume and weight as compared to negative control (Figure 7A‐C). However, we failed to identify the difference of tumour incidence between two types of grafts, although the delay of tumour onset in grafts of ZEB2‐AS1‐ASO‐treated cells was observed (data not shown). Moreover, reduced Ki67 and ZEB2 immunostaining was observed in samples derived from ASO‐ZEB2‐AS1‐transduced cells (Figure 7D). Next, we developed a systemic lung metastasis model by tail vein injection in nude mice to verify the pro‐metastatic role of ZEB2‐AS1 in vivo. As shown in Figure 7E, much fewer lung metastatic nodules were identified in ASO‐ZEB2‐AS1‐treated cell injection as compared to control cells. Together, ZEB2‐AS1 is critically involved in tumour overgrowth and metastasis of HNSCC.

## 4 DISCUSSION

The dismal prognosis of HNSCC highlights the critical need to identify more effective biomarker and druggable therapeutic targets with translational potentials.^2^ Cancerous lncRNA stands out as an optimal candidate for cancer screen, patient stratification and prognostic prediction.^4^, ^5^ Importantly, several oncogenic lncRNAs have been demonstrated with potent anti‐cancer effects when they were therapeutically inhibited in vivo.^4^, ^27^ Here, our results reveal that lncRNA ZEB2‐AS1 probably serves as pro‐oncogenic noncoding RNA by stabilizing ZEB2 mRNA and hold potentials as a novel prognostic biomarker and therapeutic target in HNSCC with its aberrant overexpression.

### 4.1 Clinical significance of ZEB2‐AS1 overexpression in HNSCC

Previous reports have delineated that ZEB2‐AS1 is aberrantly up‐regulated in several types of human cancer and associated with tumour aggressiveness and unfavourable prognosis.^16^, ^17^ For example, overexpression of ZEB2‐AS1 significantly correlated with tumour size, lymph node metastasis and tumour stage in bladder cancer.^14^ Consistent with these findings, our results from HNSCC cell lines and primary samples indicated that ZEB2‐AS1 was significantly elevated in a fraction of HNSCC as compared to their counterparts. Moreover, high ZEB2‐AS1 positively associated with larger tumour size and the presence of cervical lymph node metastasis. Kaplan‐Meir survival analysis revealed the patients with high ZEB2‐AS1 had inferior overall and disease‐free survival compared to those with low abundance of ZEB2‐AS1. Furthermore, Cox hazard regression assay identified the expression status of ZEB2‐AS1 as an independent factor affecting patient prognosis. However, limited number of patients examined and retrospective nature of our study precluded the unequivocal demonstration of prognostic significance of ZEB2‐AS1 in HNSCC. A large amount of prospectively enrolled patients is required to convincingly establish its prognostic utility in HNSCC. Collectively, our findings provide support to ZEB2‐AS1 as a novel and viable biomarker with diagnostic and prognostic values for HNSCC.

### 4.2 Tumourigenic roles of ZEB2‐AS1 in HNSCC

Accumulating evidence has shown that ZEB2‐AS1 primarily functioned as an oncogenic lncRNA in human cancer by promoting proliferation, invasion and MET and inhibiting apoptosis.^14^, ^15^, ^16^, ^17^ In lung cancer cells, knockdown of ZEB2‐AS1 inhibited cell proliferation and induced apoptosis by altering expression of Bcl‐2, Bax and caspase3/9.^16^ In addition, the effects on cell proliferation and apoptosis of ZEB2‐AS1 were probably mediated by miR‐27 in bladder cancer cell.^14^ Moreover, ZEB2‐AS1 exerted as a molecular sponge of miR‐204 and in turn derepressed miR‐204 target HMGB1 to facilitate pancreatic cancer growth and invasion both in vitro and in vivo.^15^ Generally, consistent with these abovementioned findings, our in vitro loss‐of‐function assay by ASOs revealed that depletion of ZEB2‐AS1 inhibited cell proliferation, migration and invasion, and EMT while induced apoptosis in HNSCC cell lines. Furthermore, we utilized the rhTGF‐β1‐induced EMT cellular model and found that ZEB2‐AS1 was critically involved in EMT as demonstrated that depletion of ZEB2‐AS1 largely abolished the effects of rhTGF‐β1 on EMT markers and cell invasiveness of HNSCC. This finding was in agreement with recent report, wherein TGF‐β1 secreted by cancer‐associated fibroblasts induced EMT and enhanced invasion in bladder cancer cells while depletion of ZEB2‐AS1 reversed TGF‐β1‐induced EMT and invasion.^28^ Indeed, our in vivo animal experiments offered substantial evidence to support the pro‐proliferative and pro‐metastatic roles of ZEB2‐AS1 in HNSCC. Complementary with these findings, our data also revealed that overexpression of ZEB2‐AS1 significantly associated with large tumour size and cervical node metastasis. Given the well‐established idea that cervical node metastasis is a key factor dictating patient survival, we speculate that ZEB2‐AS1 might enhance metastatic spreading and its therapeutic targeting in patients with ZEB2‐AS1 overexpression might confer clinical benefits to reduce metastasis and ultimately improve survival.

### 4.3 Mechanisms responsible for ZEB2‐AS1 in HNSCC tumourigenesis

Although the precise molecular mechanisms underlying ZEB2‐AS1 during tumourigenesis remain incompletely known, previous studies have indicated that ZEB2‐AS1 serves as molecular sponge for specific miRNA and subsequently derepresses the target of miRNA, which facilitates cancer overgrowth and dissemination.^14^, ^15^ For example, miR‐27 and miR‐204 abundance was attenuated by ZEB2‐AS1 overexpression and then increased targets of these miRNAs to modulate cancer aggressiveness in bladder and pancreatic cancer.^14^, ^15^ Considering the facts that ZEB2‐AS1 was a NAT corresponding to the 5′ UTR of ZEB2 and NAT might increase the stability of their target sense mRNAs by forming RNA duplex and masking specific sites that would otherwise resulted in mRNA degradation,^29^, ^30^ we determined the stability of ZEB2 mRNA following ZEB2‐AS1 knockdown and found that ZEB2 mRNA stability was significantly impaired following ZEB2‐AS1 inhibition and actinomycin D exposure. Moreover, our rescue experiments revealed that ZEB2 served as at least one of the downstream targets of ZEB2‐AS1 underlying its roles in EMT and metastatic dissemination. Complementarily, positive correlations between ZEB2‐AS1 and ZEB2 mRNA in HNSCC cell lines and samples, markedly reduced ZEB2 upon ZEB2‐AS1 depletion as well as similar phenotypic changes upon ZEB2 or ZEB2‐AS1 depletion provided experimental support to this regulatory model. Consistently, previous reports have shown that synthesis of ZEB2 protein is controlled by ZEB2‐AS1 via preventing splicing of ZEB2 5'‐UTR and keeping an internal ribosome entry site for its protein translation.^24^, ^25^ Noticeably, reintroduction of exogenous ZEB2 failed to significantly abrogate the impaired cell proliferation induced by ZEB2‐AS1 depletion. We speculate that other targets independent of ZEB2 responsible for ZEB2‐AS1's pro‐proliferative roles exist, which needs further experimental clarifications. Together, our findings support that the pro‐tumourigenic roles of ZEB2‐AS1 are mediated, at least in part, by ZEB2 in HNSCC.

In conclusion, our data reveal that ZEB2‐AS1 is highly expressed in a fraction of HNSCC and its overexpression significantly associates with tumour aggressiveness and unfavourable prognosis. ZEB2‐AS1 promotes EMT and invasion probably via modulating the stability of ZEB2 mRNA in HNSCC. More experimental studies have been warranted to further unravel the detailed regulatory mechanisms underlying ZEB2‐AS1 overexpression and its oncogenic roles in HNSCC.

## CONFLICT OF INTEREST

The authors declare that they have no competing interests.

## AUTHORS' CONTRIBUTIONS

PD and HG performed the experimental study, data collection and analysis and manuscript writing. PD, YS and YW carried out the most experiments. JL, ZL and JY performed histological and statistical analyses. JC and YW conceived and supervised the whole project. All authors read and approved the final manuscript.
