# Supplementary figures and images for: Overexpression of ZEB2‐AS1 promotes epithelial‐to‐mesenchymal transition and metastasis by stabilizing ZEB2 mRNA in head neck squamous cell carcinoma
Source: J Cell Mol Med. 2019 Apr 4;23(6):4269–80. doi: 10.1111/jcmm.14318 (PMC6533490; doi:10.1111/jcmm.14318)

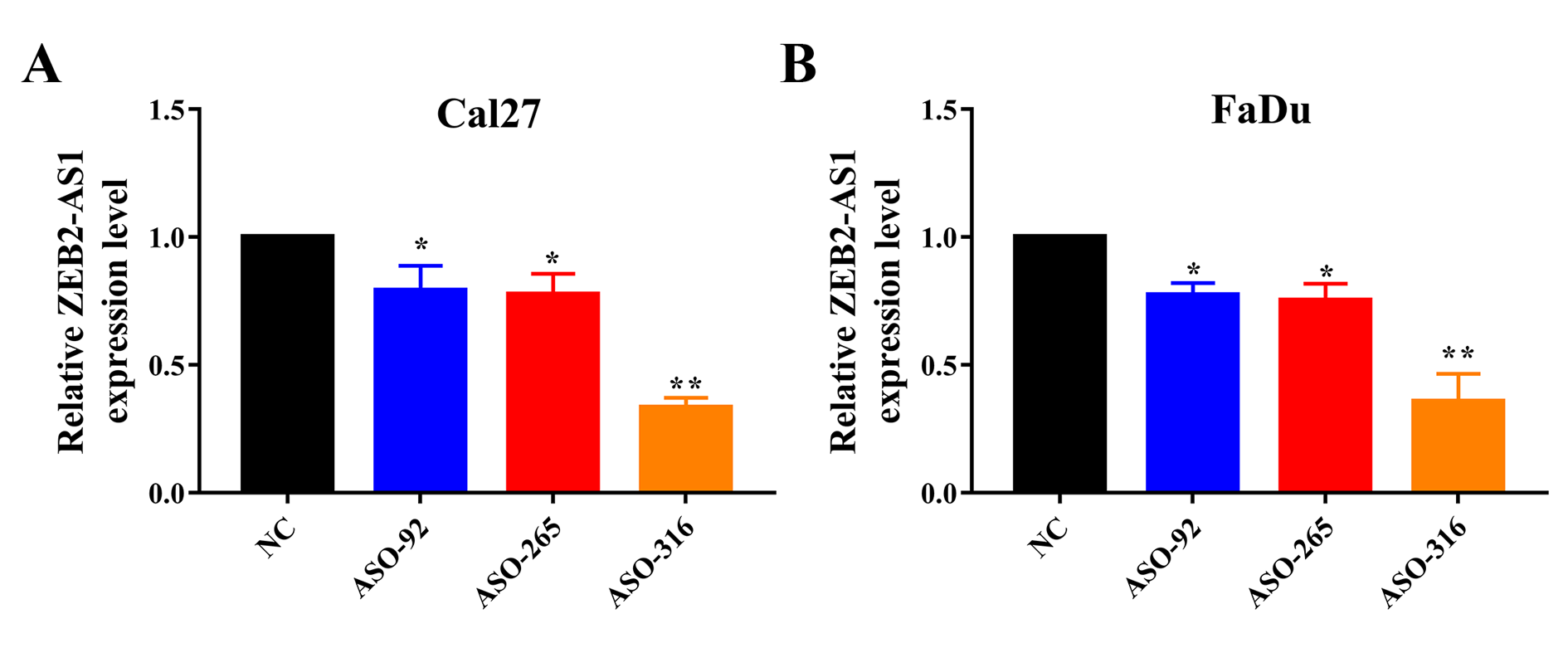

Supplement: Supplementary file 1 [file JCMM-23-4269-s001.tif]

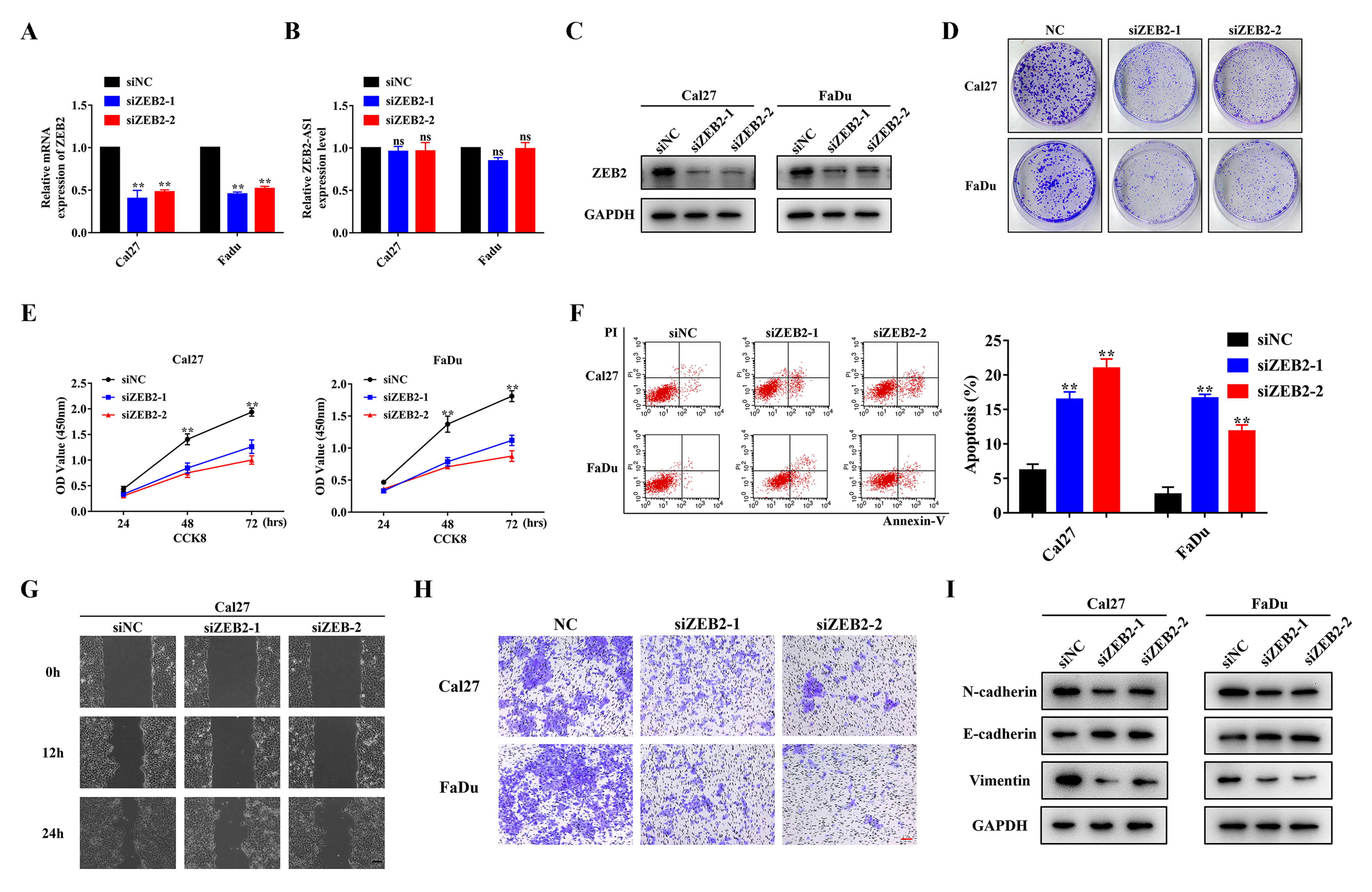

Supplement: Supplementary file 2 [file JCMM-23-4269-s002.tif]

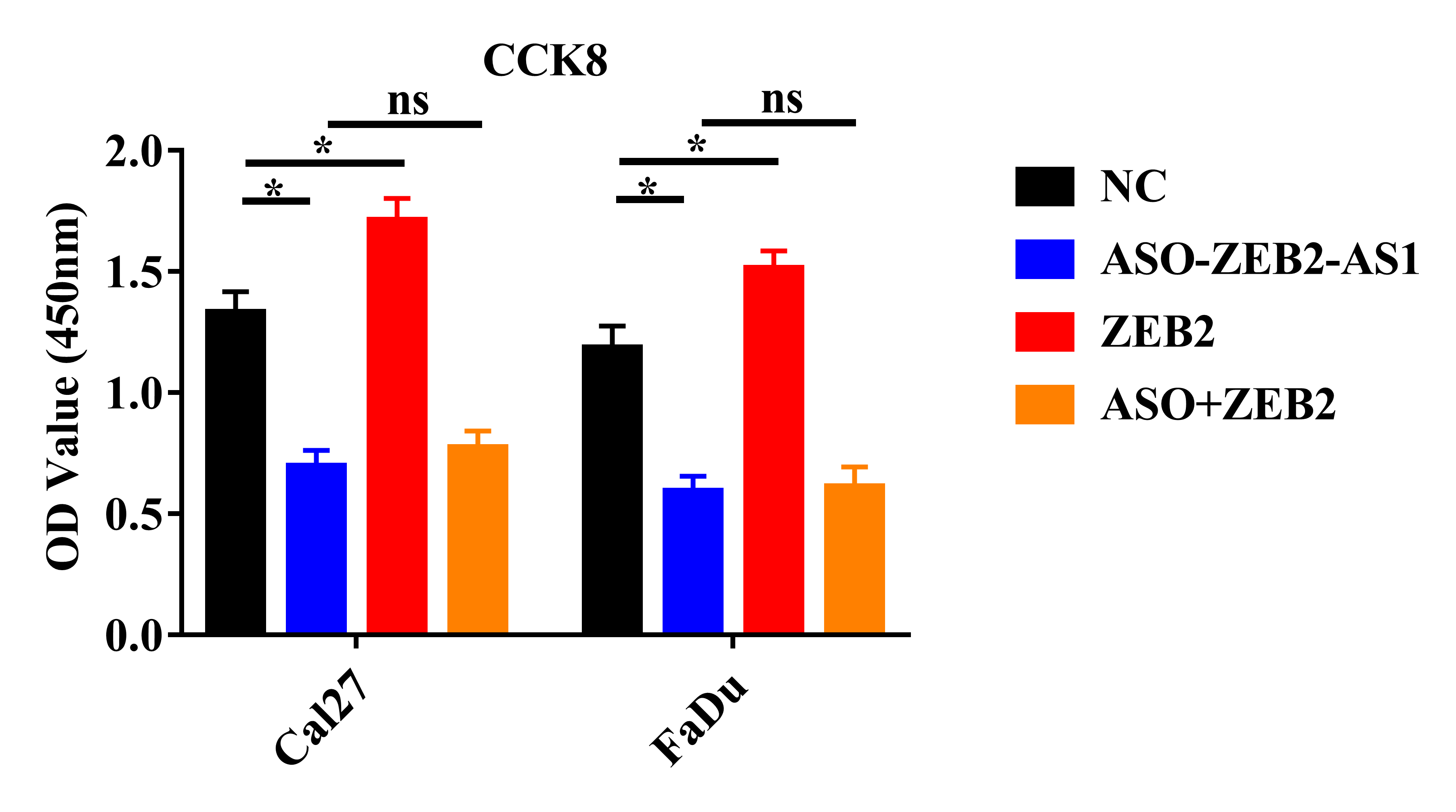

Supplement: Supplementary file 3 [file JCMM-23-4269-s003.tif]

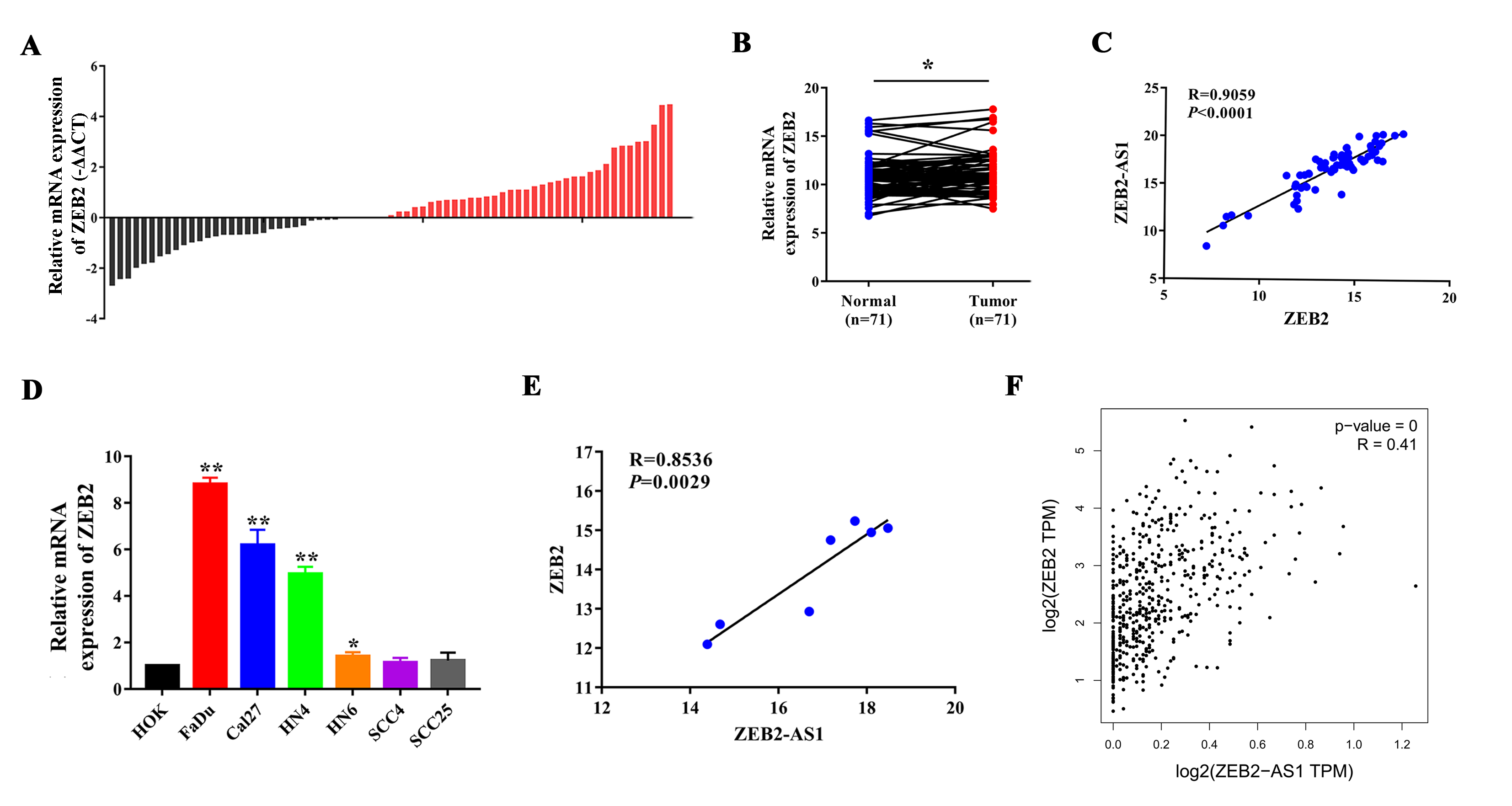

Supplement: Supplementary file 4 [file JCMM-23-4269-s004.tif]

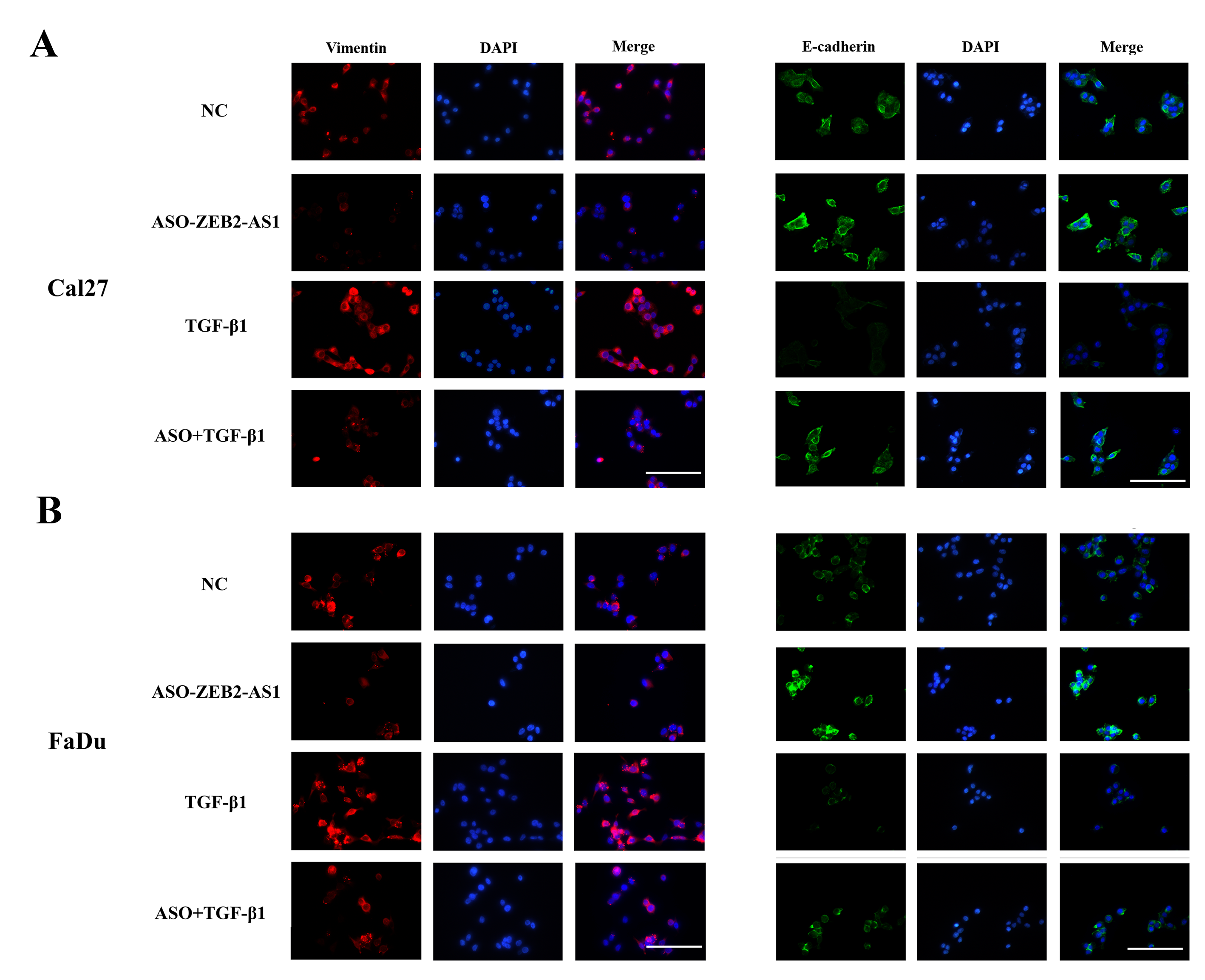

Supplement: Supplementary file 5 [file JCMM-23-4269-s005.tif]
