# Supplementary material for: Overexpression of ZEB2‐AS1 promotes epithelial‐to‐mesenchymal transition and metastasis by stabilizing ZEB2 mRNA in head neck squamous cell carcinoma
Source: J Cell Mol Med. 2019 Apr 4;23(6):4269–80. doi: 10.1111/jcmm.14318 (PMC6533490; doi:10.1111/jcmm.14318)
Supplement: Supplementary file 6 [file JCMM-23-4269-s006.docx]

**Supplementary Table 1. Correlation between ZEB2-AS1 expression and multiple clinicopathological parameters in HNSCC**

| **Clinicopathological parameters** | **ZEB2-AS1** | | ***P*-values** |
| --- | --- | --- | --- |
|  | **Low** | **High** |  |
| **No. of patients** | 36 | 35 |  |
| **Gender** |  |  |  |
| Male | 24 | 18 | 0.2318 |
| Female | 12 | 17 |  |
| **Age** |  |  |  |
| <60 | 12 | 10 | 0.7985 |
| ≥60 | 24 | 25 |  |
| **Smoking** |  |  |  |
| No | 19 | 25 | 0.1435 |
| Yes | 17 | 10 |  |
| **Alcohol use** |  |  |  |
| No | 22 | 26 | 0.3121 |
| Yes | 14 | 9 |  |
| **Tumor size** |  |  |  |
| T1-T2 | 27 | 17 | **0.0287** |
| T3-T4 | 9 | 18 |  |
| **Pathological grade** |  |  |  |
| Ⅰ | 26 | 19 | 0.1435 |
| Ⅱ-Ⅲ | 10 | 16 |  |
| **Cervical node metastasis** |  |  |  |
| N(0) | 25 | 15 | **0.0321** |
| N(+) | 11 | 20 |  |
| **Clinical stage** |  |  |  |
| Ⅰ-Ⅱ | 22 | 23 | 0.8065 |
| Ⅲ-Ⅳ | 14 | 12 |  |

The number in bold indicate statistical significance with *P*-values less than 0.05.
