# Supplementary material for: Overexpression of ZEB2‐AS1 promotes epithelial‐to‐mesenchymal transition and metastasis by stabilizing ZEB2 mRNA in head neck squamous cell carcinoma
Source: J Cell Mol Med. 2019 Apr 4;23(6):4269–80. doi: 10.1111/jcmm.14318 (PMC6533490; doi:10.1111/jcmm.14318)
Supplement: Supplementary file 7 [file JCMM-23-4269-s007.docx]

| **Supplementary Table 2.**  **Univariate and Multivariate Cox-regression analyses of ZEB2-AS1 and clinicopathological parameters in HNSCC** | | | | | |
| --- | --- | --- | --- | --- | --- |
| **Variables** | **Univariate analyses** | |  | **Multivariate analyses** | |
|  | **HR [95% CI]** | ***P*** |  | **HR [95% CI]** | ***P*** |
| **Overall survival** |  |  |  |  |  |
| Age (≥60, <60) | 0.752(0.332-1.703) | 0.494 |  | 0.736(0.306-1.771) | 0.494 |
| Gender (male, female) | 0.745(0.339-1.637) | 0.464 |  | 0.392(0.119-1.291) | 0.124 |
| Smoking (Yes, No) | 1.211(0.544-2.697) | 0.639 |  | 2.482(0.814-6.632) | 0.348 |
| Alcohol use (Yes, No) | 1.018(0.439-2.358) | 0.968 |  | 0.565(0.120-2.656) | 0.470 |
| Tumor size (T3-T4, T1-T2) | 2.550(1.155-5.631) | **0.021** |  | 2.172(0.741-6.364) | 0.157 |
| Pathological grade (III-IV, I) | 1.342(0.608-2.964) | 0.466 |  | 0.577(0.197-1.685) | 0.315 |
| Cervical nodal metastasis (N+, N0) | 1.694(0.772-3.719) | 0.189 |  | 1.969(0.673-5.762) | 0.216 |
| Clinical stage (III-IV, I-II) | 1.247(0.560-2.778) | 0.589 |  | 0.924(0.328-2.604) | 0.881 |
| ZEB2-AS1 expression (High, Low) | 2.724(1.174-6.321) | **0.020** |  | 3.113(1.082-8.957) | **0.035** |
| **Disease-free survival** |  |  |  |  |  |
| Age (≥60, <60) | 0.918(0.434-1.941) | 0.822 |  | 0.848(0.377-1.903) | 0.689 |
| Gender (male, female) | 0.647(0.322-1.299) | 0.220 |  | 0.322(0.209-1.555) | 0.141 |
| Smoking (Yes, No) | 1.075(0.525-2.202) | 0.842 |  | 2.545(0.932-5.171) | 0.261 |
| Alcohol use (Yes, No) | 0.984(0.466-2.080) | 0.967 |  | 0.744(0.173-3.203) | 0.692 |
| Tumor size (T3-T4, T1-T2) | 2.272(1.132-4.560) | **0.021** |  | 1.963(0.774-4.980) | 0.155 |
| Pathological grade (III-IV, I) | 1.099(0.540-2.236) | 0.795 |  | 0.468(0.182-1.205) | 0.116 |
| Cervical nodal metastasis (N+, N0) | 1.506(0.751-3.022) | 0.249 |  | 2.076(0.822-5.246) | 0.122 |
| Clinical stage (III-IV, I-II) | 1.289(0.636-2.613) | 0.481 |  | 1.119(0.453-2.767) | 0.808 |
| ZEB2-AS1 expression (High, Low) | 2.479(1.193-5.149) | **0.015** |  | 2.885(1.138-7.315) | **0.026** |
| HR, hazard ratio; CI, confidence interval. |  |  |  |  |  |
